# The impact of family function on mental health status in patient with inflammatory bowel disease: The mediating role of self-esteem

**DOI:** 10.3389/fpsyt.2022.1007318

**Published:** 2022-12-01

**Authors:** Qiwei Wu, Pingting Zhu, Xinyi Liu, Chen Chen, Qiaoying Ji, Qiaohua Gu

**Affiliations:** ^1^School of Nursing and Public Health, Yangzhou University, Yangzhou, China; ^2^Gastroenterology Department, Northern Jiangsu People's Hospital, Yangzhou, Jiangsu, China

**Keywords:** inflammatory bowel disease, family function, self-esteem, depression, anxiety, mediating

## Abstract

**Objective:**

Family function is a protective factor for mental health status in IBD patients; however, the underlying processes are unknown. This study aimed to investigate the mediating influence of self-esteem on family functioning and mental health.

**Methods:**

This cross-sectional study comprised a total of 133 IBD patients who were assessed for family function (APGAR) and depression symptoms. (PHQ-9), anxiety symptoms. (GAD-7) and self-esteem (RSES) *via* self-administered questionnaires. Mediating effects were tested using the SPSS Process program with bootstrap.

**Results:**

The total score of PHQ-9 was 7.44 ±5.54. The total score of GAD-7 was 6.15±4.78. Significant associations were identified among family function, self-esteem, depression, and anxiety symptoms. Results revealed a significant indirect effect, suggesting that the effects of family function on depression and anxiety symptoms were mediated by self-esteem; the indirect effects percentages were 41.63 and 29.25.

**Conclusion:**

These results indicate that the family function of IBD patients can predict their mental health condition. As self-esteem is a mediating element, which may have been influenced by family function, mental health status is indirectly affected. Therefore, promoting the self-esteem of IBD patients is crucial for facilitating long-term mental wellness.

## Introduction

Inflammatory bowel diseases (IBD), which include Crohn's disease (CD) and ulcerative colitis (UC), are chronic and recurrent gastrointestinal illnesses that cause inflammation (1). The prevalence of IBD has increased globally due to changing environmental factors including westernized diets, socio-economic changes (2). With advances in illness knowledge and diagnostic technology, the number of individuals identified with inflammatory bowel disease (IBD) has steadily grown, hence increasing the economic burden of IBD (3). Although medications and treatment procedures for IBD are continually being updated and improved, at the current medical level, all that can be accomplished is to extend the remission duration and prognosis of the disease (4). However, IBD is defined by a protracted lifelong course of disease and recurrence, as well as several comorbidities, which frequently result in patients falling into a condition of poor mental health, negatively impacting their quality of life and social abilities. According to a comprehensive study and meta-analysis, the prevalence of anxiety or depressive symptoms in IBD patients was 32.1 and 25.2%, respectively (5). In addition, bidirectional communication *via* the gut-brain axis, the basis of the psychophysiological susceptibility of IBD patients, may have a detrimental impact on IBD patients' symptoms, resulting in frequent hospitalizations or high disability rates (6, 7). In other words, a poor state of mental health can imprison people in an ongoing cycle of sickness. Consequently, it is crucial to identify the variables that impact IBD patients' mental health status and discover measures to enhance mental health status.

Sociodemographic factors, such as gender, age, and income; clinical factors, such as illness type and disease activity; and social factors, such as family function and social support, all influence the mental health status of IBD patients (8, 9). Individuals' family function is determined by their ability to communicate with family members, fill family roles, accept routines and procedures, manage family stress, and measure family relationships with others (10). Family is regarded as the smallest unit of society, and according to the Chinese cultural notion, it is based on blood connections and family ethics and has complimentary features. Patients may thus find their families to be the most accessible source of social support. Research has demonstrated that flourishing family functioning can enhance patient self-efficacy, increase positive coping strategies, and reduce negative psychological states (11, 12). Thus, the family function may be a protective factor for the prognosis of IBD patients, influencing their mental and physical health. In addition, the family function can bring about continuous optimization of prognosis, improve the sense of happiness, and attain a long-term s‘ quality of life (13, 14), which supports the notion that family function can bring about continuous optimization of prognosis. Research shows that family function might impact a patient's mental health. However, earlier research on the effect of family function on the mental health of IBD patients failed to establish the particular mechanism behind this association.

Self-esteem is viewed as an internal psychological resource, defined as the entire evaluation of a person's value. Its composition is founded on the notion of self and self-cognition (15). It is abundantly established that it can enhance the quality of life (16). Patients with greater levels of self-esteem may feel more confident and achieve various life objectives to increase their degree of self-recognition, social acknowledgment, and social acceptability, so improving their quality of life. According to a prior study, the stated emotional climate of a patient's Family (a measure of family emotional climate) moderated the development and expression of paranoid and positive symptoms of early psychosis (17, 18). A study in Taiwan that explored the relationship between family adversity and social anxiety in adolescents found that a decline in family functioning decreased self-esteem (19). Parental conflict is negatively correlated with adolescents' self-esteem (20). In addition, poor family function has been found in patients with substance use disorders. As a consequence of feeling unsupported by their families, they develop low self-esteem and negative self-perceptions, increasing their likelihood of relapsing (21). In addition, self-esteem is considered a protective factor for the prognosis of IBD, and greater levels of self-esteem are connected with adopting healthier practices more frequently. Similarly, high self-esteem can respond positively to stressful circumstances, lessen the stress of disease, and adapt their mental health (15). Therefore, for quality of life and prognosis, it is crucial to identify the mediation role of self-esteem between family functioning and mental health status in IBD patients. In this study, we evaluated the role of self-esteem as a mediator, which may explain the association between family function and mental health in IBD patients. We hypothesized, based on the preceding information, that (H1) family function is related to mental health status; (H2) family function is related to self-esteem; (H3) self-esteem is related to mental health status, and (H4) self-esteem mediates the relationship between family function and mental health status.

## Methods

### Study design and participants

The study adopted a cross-sectional and correlational design. This cross-sectional correlational study was conducted between October 2020 and May 2021 at two hospitals in XX with IBD patients. Participants were recruited using a convenience sample. The selection of IBD patients was based on the following criteria: (a) patients who have been diagnosed with IBD (including CD and UC); (b) patients who are at least 18 years old; (c) patients who are aware of their diagnosis; (d) patients with everyday awareness, hearing, and eyesight; (e) patients who understand the goal of the research and agree to participate. Exclusion criteria included: (a) patients with malignant tumors or other anorectal illnesses; (b) patients with known concomitant psychiatric disorders and those undertaking psychotherapy (including medication); and (c) patients with severe mental illness who were unable to comply.

We invited 146 patients who satisfied the study's inclusion criteria to participate. A total of 140 patients participated in this trial. Questionnaires that were omitted or filled in with all the same options (including reverse questions) were removed after verification by QW and PZ In the end, we obtained 133 valid questionnaires. For individuals who declined to participate, the following explanations apply: Lack of interest in the study (*n* = 3); (ii) Reluctance to be disturbed (*n* = 2); (iii) Fear of personal data leaking (*n* = 1). This study was done following the Declaration of Helsinki of the World Medical Association. Before the beginning of the trial, informed consent was acquired from every participant. The Ethics Committee of XX (XX) granted the ethical study approval.

### Measurement of variables

#### Demographic information

Included in the demographic data were gender, age, disease type, medical insurance, education level, marital status, work status, address, and monthly income.

#### Family function

We Evaluated Family Function Using the Family APGAR, Which Indicated the Individual's Subjective Sentiments About the Family and the Family's Concerns. Five Dimensions Comprise Family APGAR: Adaptation (A), Partnership (P), Growth (G), Affection (A), and Resolve (R). It Has five Questions on a 3-Point Likert Scale Ranging From 0 (Rarely) to 2 (Very Often). Total Scores of 0–3, 4–6, and 7–10 Indicate Poor, Moderate, and Excellent Family Functioning, Respectively. The Chinese Version of the Family APGAR Has Been Utilized Extensively due to Its High Validity and Reliability (22, 23). In This Research, Cronbach's α of the Scale Was 0.795.

#### Mental health statuses

##### The generalized anxiety disorder 7-item scale

The Generalized Anxiety Disorder 7-Item Scale (GAD-7) was used to identify symptoms of generalized anxiety disorder according to the DSM-IV. The GAD-7 is a seven-item self-report measure used to assess the severity of anxiety symptoms during the past 2 weeks. The items are scored on a 4-point Likert scale (ranging from 0 = hardly at all to 3 = virtually every day), and the total score ranges from 0 to 21. Five represents mild symptoms, 10 indicates moderate symptoms, and 15 suggests severe anxiety symptoms (24). Earlier investigations have confirmed the validity and reliability of the GAD-7 Chinese version instrument (25). The Cronbach's α coefficient of the GAD-7 in this study was 0.901.

##### The patient health questionnaire

The Patient Health Questionnaire (PHQ-9) was employed to identify symptoms of major depression. This scale is measured on a 4-point Likert scale (ranging from 0 = hardly at all to 3 = virtually every day) with a total score range of 0 to 27. The total scores of 5–9, 10–4, 15–19, and 20–27 reflect mild, moderate, moderately severe, and severe depressive symptoms, respectively (26). The Chinese version PHQ-9 has demonstrated validity and reliability (Cronbach's α = 0.938) (27), and is also a reliable measure of depression symptoms. In this study, the Cronbach's coefficient for the PHQ-9 was 0.887.

#### Self-esteem

The self-esteem was evaluated using Rosenberg Self-Esteem Scale (RSES). It consists of 10 items with a four-point response scale (1 = strongly disagree; 2 = disagree; 3 = agree; 4 = strongly agree). The overall score ranges from 0 to 30 points. Stronger scores imply higher self-esteem levels. Internal consistency has been high in a lot of research on Chinese people (28). Cronbach's alpha for this study is 0.798, indicating that it has a high level of reliability.

### Statistical analyses

For statistical analysis, SPSS (Version 21) was employed. Mean, standard deviation (MSD), and percentages were used to present descriptive data and demographic information. The *T*-test and ANOVA were used to assess the differences between the PHQ-9 and GAD-7 scores. Depending on the context, the Pearson product-moment or the Spearman rank coefficient was used to investigate the relationships between the principal variables. Based on previous studies and in order to test our hypotheses, we took family function as the independent variable and indicators reflecting mental health status (PHQ-9 and GAD-7) as the dependent variable. To explore the relationship between family function and mental health status, we used self-esteem as a mediating variable. In addition, all models controlled for covariates (variables significant for the dependent variable in demographic data) and standardized the study variables. The bias-corrected bootstrap technique utilizing the SPSS Process tool (Model 4) was used to assess mediating effects. Using ordinary least squares regression, the PROCESS mediation analysis is performed. Control variables such as illness type, education level, and place of residence were introduced in the model as covariates. We calculated the interval using 5,000 bootstrap samples with retraction and establishing a 95% confidence interval for the value of the mediating effect. The importance of the mediating impact was determined by excluding 0 from the top and lower boundaries of the interval, which means the indirect and conditional influence were deemed significant and the self-esteem can be recognized as a mediating variable.

## Results

### Demographic information and mental health status

Table 1 displays the demographic statistics of the 133 Chinese participants with IBD. The mean age was 33.13 years with a standard deviation of 12.71 years. Men comprised more than half of the participants (57.1%). Most were diagnosed with UC (72.2%) and had a high school diploma or less (67.7%). In total of 72.9% of patients had medical insurance coverage. In addition, just 48.1% of participants were getting married. In total of 65.4% reported being employed. A fifth of the state's participants alternate work/school with sick leave (28.6%). The majority of them reside in rural regions (55.7%). The full PHQ-9 score was 7.44 ± 5.54, and the total GAD-7 score was 6.15 ± 4.78. We used a score of eight to determine depression, and 61 (45.9%) patients met the cut-off value for the diagnosis of depression (29). After setting score 10 as the cut-off value for the GAD-7, 33 (24.8%) patients were considered to have anxiety (30). The independent sample *T*-test and ANOVA revealed that illness type, education level, and residence were substantially associated with patients' depressive and anxious symptoms, respectively.

**Table 1:** Mental health status by demographic information.

**Demographic information**	***N* (%)**	**PHQ-9**		**GAD-7**	
		***t*/F**	***P***	***t*/F**	***P***
Age		0.528^a^	0.591	0.379^a^	0.685
< 18	5 (3.7)				
18–45	98 (73.7)				
>46	30 (22.6)				
Gender		−1.259^b^	0.210	−0.381^b^	0.704
Male	76 (57.1)				
Female	57 (42.9)				
Disease type		−2.293^b^	0.023^*^	−2.497^b^	0.014^*^
Crohn's disease	96 (72.2)				
Ulcerative colitis	37 (27.8)				
Medical insurances		−1.079^b^	0.282	−0.189^b^	0.850
No	36 (27.1)				
Yes	97 (72.9)				
Education level		2.911^a^	0.037^*^	2.889^a^	0.038^*^
Primary school and below	3 (2.2)				
Middle school and below	39 (29.4)				
High or vocational school	48 (36.1)				
College and above	43 (32.3)				
Marital status		−0.543^b^	0.588	0.376^b^	0.708
Married	64 (48.1)				
Unmarried/Divorced/Widowed	69 (51.9)				
Employment status		0.496^a^	0.610	1.161^a^	0.316
Employed	49 (36.8)				
Alternate work/school with sick leave	38 (28.6)				
Unemployed	46 (34.6)				
Residence		3.173^a^	0.045^*^	1.172^a^	0.313
Urban	59 (44.3)				
County	36 (27.1)				
Rural	38 (28.6)				
Monthly salary of individual (EUR)		1.275^a^	0.286	1.101^a^	0.351
< 145	23 (17.4)				
145 ~ 285	22 (16.5)				
286 ~ 715	49 (36.8)				
≥ 715	39 (29.3)				

a = F value;

b = *t* value;

* *P* < 0.05.

### Correlation between family function, mental health status and self-esteem

As demonstrated in Table 2, the mean score for GAD-7, PHQ-9, self-esteem, and family function were 6.15 (SD 7.48), 7.44 (SD 5.54), 27.96 (SD 4.53), and 6.36 (SD 2.24), respectively. Both GAD-7 and PHQ-9 were adversely linked with self-esteem (*r* = −0.527, *P* <0.01; *r* = −0.602, *P* <0.01) and family function (*r* = −0.532, *P* <0.01; *r* = −0.506, *P* <0.01). The relationship between self-esteem and family function is favorable (*r* = 0.427, *P* <0.01).

**Table 2:** The scores of main variables and correlation between family function, mental health status and self–esteem (*N* = 133).

	**Mean (SD)**	**GAD7**	**PHQ9**	**Self-esteem**	**Family**
GAD-7	6.15 (4.78)	1			
PHQ-9	7.44 (5.54)	0.747^**^	1		
Self-esteem	27.96 (4.53)	−0.527^**^	−0.602^**^	1	
Family function	6.36 (2.24)	−0.532^**^	−0.506^**^	0.427^**^	1

** *P* < 0.01.

### Mediation of self-esteem in the relationship between family function, mental health status

Indicators of mental health status (PHQ-9 and GAD-7) were chosen as independent factors, whereas family support was chosen as a dependent variable. In the mediation analysis, disease type, education level, and residence were input as covariates.

**Figure 1:**
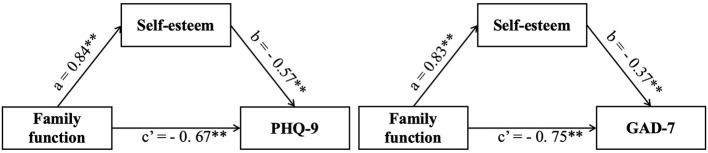
Mediating role of self-esteem on the relationships between family function and mental health status.

First of all, after controlling for illness type, education level, and place of residence, the effect of family function on PHQ-9 and GAD-7 of IBD patients were analyzed. The total impact was significant (*B* = −1.15, *P* <0.01; *B* = −1.05, *P* <0.01). Then, the intermediary role of self-esteem in the influence of family function on PHQ-9 and GAD-7 were analyzed. In this step, family function had significant positive and direct effect on depressive symptoms and anxiety symptoms (*B* = 0.84, *P* <0.01, 95%; *B* = 0.83, *P* <0.01). After controlling for variables, the effect of self-esteem on depression and anxiety symptoms (*B* = −0.57, P.01, 95%; *B* = −0.37, *P* <0.01) was similarly significant. Furthermore, the indirect effect size was calculated using the formula (|a path ^*^ b path/c path|) to determine the predicted proportion of self-esteem in the model. The indirect effect of self-esteem on depression and anxiety symptoms accounts for 41.63 and 29.25%, respectively, indicating that family function can impact mental health status through self-esteem (see Tables 3, 4). From the results above, family function was directly related to PHQ-9 and GAD-7, and it can also affect them through the mediation of self-esteem (see Figure 1).

**Table 3:** Mediating effect of self-esteem on the relationship between the family function and PHQ-9 (*N* = 133).

	***R***	***R*-sq**	***F***	**Coefficient**	**SE**	***t***	**95% CI**	
							**LLCI**	**ULCI**
FF→ PHQ-9 (c path)	0.55	0.30	13.61^**^	−1.15	0.19	−6.16^**^	−1.52	−0.78
FF→ SE (a path)	0.46	0.21	8.43^**^	0.84	0.16	5.19^**^	0.52	1.17
SE→ PHQ-9 (b path)	0.68	0.47	22.41^**^	−0.57	0.09	−6.38^**^	−0.74	−0.39
FF→ PHQ-9 (c' path)				−0.67	0.18	−3.75^**^	−1.13	−0.32

FF, family function; SE, self-esteem; SE, standard error; CI, confidence interval; LLCI, lower level of confidence interval; ULCI, upper level of confidence interval;

** *P* < 0.01.

**Table 4:** Mediating effect of self-esteem on the relationship between the family function and GAD-7 (*N* = 133).

	***R***	***R*-sq**	***F***	**Coefficient**	**SE**	***t***	**95% CI**	
							**LLCI**	**ULCI**
FF→ GAD-7 (c path)	0.56	0.32	19.88^**^	−1.05	0.16	−6.64^**^	−1.37	−0.74
FF→ SE (a path)	0.44	0.19	10.06^**^	0.83	0.16	5.06^**^	0.50	1.15
SE→ GAD-7 (b path)	0.64	0.42	22.77^**^	−0.37	0.08	−4.67^**^	−0.53	−0.21
FF→ GAD-7 (c' path)				−0.75	0.16	−4.64^**^	−1.07	−0.43

** *P* < 0.01.

## Discussion

This study aims to explore mental health status and elucidate the interrelationships between family function and mental health status among IBD patients in China while considering self-esteem. There was a strong correlation between low family function and high mental health status among IBD patients. Furthermore, we discovered that self-esteem moderated the impact of family function on mental health status.

The full scores for PHQ-9 and GAD-7 were similar to those previously seen in Chinese patients with IBD, but higher than in other countries and regions (31, 32). It might be connected to the participant selection procedure. Since all of the participants were recruited during hospitalization, the acute phase of the disease and the inpatient setting may exacerbate the participants' anxiety and sadness. Xu's study also showed that anxiety and depression were significantly higher during the active phase of the disease (32). A lack of financial and medical support for people with inflammatory bowel disease in China may also contribute to their poor mental health (33). In this study, anxiety and depression levels are associated to the patient's condition, degree of education, and place of living. Regarding illness categories, GAD-7 and PHQ-9 scores were recorded for CD (5.52 ± 4.62, 6.77 ± 5.29) and UC (7.78 ± 4.86, 9.19 ± 5.86), respectively.

On the one hand, it may result from the disease's impact on UC patients. In Mandel's investigation of hospitalization rates among IBD patients, the risk of IBD-related hospitalization fell only in CD patients receiving the same treatment (anti-TNF medication) (34). In contrast, UC patients demonstrate more significant extraintestinal symptoms than CD patients before the beginning of IBD (35). Due to their earlier exposure to extraintestinal signs, UC patients may experience physical and psychological suffering. On the other hand, it may be a result of the increased financial stress UC patients endure. Currently, only CD patients are insured in China, substantially raising the financial burden on UC patients (36). Currently, UC and CD are treated identically, meaning that individuals with UC are more financially pressured owing to Lack of health insurance (37). In addition, work absenteeism and work impairment due to sickness are significant contributors to patients' financial stress. Even though cohort research revealed that CD has a slightly greater incidence of work impairment than UC (compared to the general population, the RR for CD and UC were 2.0 and 1.8, respectively) (38), patients with UC may confront a more challenging economic condition, which may deteriorate their mental health, given that the existing medical insurance in China favors CD. Even though the prevalence of IBD is rising year, it is still a relatively uncommon disease in China. Therefore, the patient's degree of knowledge will impact their acceptance and understanding of this rare condition.

Additionally, only metropolitan hospitals offer tailored therapy. For instance, the availability of some specialized medications (e.g., mesalazine, infliximab) and treatment techniques (eg., fecal transplantation). Patients from rural areas must travel back and forth to receive therapy, which raises their financial and psychological burdens. Because there are still issues in the differential diagnosis of IBD and extraintestinal symptoms may predate the time of diagnosis (39), we did not limit the duration of IBD throughout the recruiting procedure. A comprehensive review and meta-analysis indicated that only five of the 11 included research found a correlation between a depressed state and disease progression (40).

We analyzed family function, and the overall score was 6.36 ± 2.24, indicating that, in general, the family function of IBD patients we studied is mild to moderately dysfunctional. This may be owing to the high expenditures on sickness treatment, which have exacerbated family tensions and led to dysfunctional households (36). According to Dibley's research (41), the stigma associated with kinship and the unfavorable views of family members affects family functioning. Furthermore, familial stigma has a more significant harmful impact than other kinds of stigma (41). Furthermore, stigma has various aspects since it will be stigmatized by the public and family members (42, 43). Our study demonstrated a substantial negative connection between family function, anxiety, and depression symptoms (44). In IBD patients, family functioning is essential to mental health improvement. Families are viewed as the most acceptable source of social support in Chinese culture because of the links of blood ties. By offering psychological and behavioral support and training family members on problem-solving approaches and tactics, patients with IBD can lower the chance of developing a poor mental state. This is congruent with the family ecosystem hypothesis, which asserts that the family system significantly impacts the psychological development of family members (45). Consequently, the higher the degree of family function, the greater the level of family members' physical and mental health and adaptation (46).

Comparable to other studies (15) the average self-esteem score among IBD patients in our study is 27.96 ± 4.53. This study demonstrates that family function positively predicts self-esteem. A higher level of family function increases one's self-esteem. A bad family environment might harm the patient's recovery because family care makes the sufferer feel valued and involved. The study also demonstrates a negative correlation between self-esteem, anxiety, and depressive symptoms. In addition, depressive and anxiety disorders often co-occur with IBD, showing that self-esteem is connected to anxiety and depression symptoms. Multiple ideas postulate that self-esteem may serve as a buffer against anxiety and depressive symptoms. According to the vulnerability model, based on the diathesis-stress framework, negative self-evaluations are a risk factor for developing depressive symptoms (47, 48). In addition, the tripartite model predicted that depression symptoms would have a greater relationship with self-esteem than anxiety symptoms, which was supported by the findings of this study (49, 50). It suggests that a boost in self-esteem may be a preventive measure that minimizes anxiety and depression symptoms.

This is the first study to elucidate how family functioning influences the mental health status of IBD patients. This may provide future insight on how to enhance the mental health of IBD patients. The most notable finding was that self-esteem significantly mediated the relationship between family function and mental health status. This suggests that boosting a patient's self-esteem may enhance their mental health, such as through cognitive behavior therapy or human imagery intervention (51, 52). IBD patients experience uncomfortable bowel symptoms (e.g., intestinal sounds, farting, bowel urgency). Those who get specific therapies frequently experience changes in appearance (e.g., wearing a nasogastric tube or developing a fistula) and limited social adaptation, resulting in low self-esteem. From a social psychology viewpoint (15), the poor self-esteem of IBD patients might result in unpleasant feelings and low life satisfaction (53). Studies have demonstrated that families may give physical and mental comfort to ailing family members through information, reward, emotional, and tool support (54). Therefore, IBD patients will feel appreciated, needed, and cared for by their family members, which will boost their sense of self-worth. This study's model of the mediating impact elucidates the significance of self-esteem in the transfer of family function and the development of a high-level mental health condition. Notably, healthcare providers should be aware that family-centered therapies might enhance the mental health status of IBD patients by fostering self-esteem.

Even regular interaction with the patient is insufficient for family members to comprehend the disease's complexities (55). Therefore, most family members tend to disregard the psychological sentiments of patients while assisting them with food and behavior management. However, despite feeling supportive, the Family may unintentionally stigmatize the individual with IBD. Since incorrect or excessive caring may have a detrimental effect on self-esteem. Healthcare professionals (HCPs) should be aware that family-centered treatments might enhance the mental health of IBD patients by enhancing their self-esteem. Helping patients with IBD by normalizing illness within the Family can benefit HCPs. Chronically sick people may interpret the normalization of a family as minimizing or hiding their condition. For instance, the dyadic communication intervention for sickness comprises family members' psychological adjustment and support techniques (56). Since IBD is a chronic condition, the familial adaption process will continue to advance. Understanding the context of IBD for the patient and Family might be facilitated by familiarity with unique patient difficulties. Enhancing the Family's internal environment is conducted in the context of the patient's overall care. Financial toxicity of illness treatment was prevalent, resulting in cost-related pharmaceutical non-adherence or even previous therapy to not add to the Family's financial burden (57). Consequently, consideration should be given to implementing medical insurance coverage for IBD patients while coping with economic difficulties or when employment serves as a buffer or diversion from financial stress.

Facing an expanding IBD population. Understanding the association between family function and mental health status might help HCPs identify areas that may need to be addressed to improve IBD patients' prognosis. In addition, it is required to design a method for measuring family function that can be used to IBD patients while taking the self-esteem dimension into account so that precise intervention measures may be taken. Significantly, considerable improvements will only result if these results are recognized by treatments and implemented in the clinic.

## Limitations

Several of our study's shortcomings must be addressed. First, because the participants were recruited from a particular location and the sample size was small, it is difficult to generalize the results to all patients with IBD. Second, because we utilized a cross-sectional approach, the causal relationship between family function and mental health status cannot be established, necessitating further longitudinal study. In conclusion, all research relies on self-report questionnaires, which may be biased owing to individual variations (eg., comprehension ability).

## Conclusions

In conclusion, we have revealed in this study that poor mental health conditions, such as anxiety symptoms or depressive symptoms, in people with IBD can be predicted by family function. In addition, self-esteem modulates the impacts between family function and mental health status, enhancing our comprehension of their underlying processes. A poor condition of family functioning among IBD patients might also be a factor in determining their mental health status. Therefore, it is a practical technique for HCPs to increase the internal function of the Family, enhance self-esteem, and promote mental health.

## Data availability statement

The data analyzed in this study is subject to the following licenses/restrictions: Raw data can be requested from the corresponding author upon request. Requests to access these datasets should be directed to PZ, ptzhu@yzu.edu.cn.

## Ethics statement

The studies involving human participants were reviewed and approved by Ethics Committee of School of Nursing, Yangzhou University (YZUHL2021008). Written informed consent to participate in this study was provided by the participants' legal guardian/next of kin.

## Author contributions

Conceptualization, methodology, and validation: PZ and QW. Software: XL. Formal analysis: XL and QJ. Investigation: QW, CC, QJ, and QG. Writing—original draft preparation: QW. Writing—review and editing and supervision: PZ. All authors have read and agreed to the published version of the manuscript.

## Funding

This study was supported by Postgraduate Research and Practice Innovation Program of Jiangsu Province (SJCX21_1653), Science and Technology Planning Project of Yangzhou (YZ2021064), and Yangzhou University International Academic Exchange Fund.

## Conflict of interest

The authors declare that the research was conducted in the absence of any commercial or financial relationships that could be construed as a potential conflict of interest.

## Publisher's note

All claims expressed in this article are solely those of the authors and do not necessarily represent those of their affiliated organizations, or those of the publisher, the editors and the reviewers. Any product that may be evaluated in this article, or claim that may be made by its manufacturer, is not guaranteed or endorsed by the publisher.
